# Heterologous expression of cobalamin dependent class-III enzymes

**DOI:** 10.1016/j.pep.2020.105743

**Published:** 2021-01

**Authors:** Tom Halliwell, Karl Fisher, Karl A.P. Payne, Stephen E.J. Rigby, David Leys

**Affiliations:** a Manchester Institute of Biotechnology, University of Manchester, 131 Princess Street, Manchester, M1 7DN, UK; b Future Biomanufacturing Research Hub (FutureBRH), Manchester Institute of Biotechnology, University of Manchester, 131 Princess Street, Manchester, M1 7DN, UK

**Keywords:** Reductive dehalogenase, Organohalide respiration, Epoxyqueuosine reductase, EPR, Cobalamin, Fe–S cluster

## Abstract

The family of cobalamin class-III dependent enzymes is composed of the reductive dehalogenases (RDases) and related epoxyqueuosine reductases. RDases are crucial for the energy conserving process of organohalide respiration. These enzymes have the ability to reductively cleave carbon-halogen bonds, present in a number of environmentally hazardous pollutants, making them of significant interest for bioremediation applications. Unfortunately, it is difficult to obtain sufficient yields of pure RDase isolated from organohalide respiring bacteria for biochemical studies. Hence, robust heterologous expression systems are required that yield the active holo-enzyme which requires both iron-sulphur cluster and cobalamin incorporation.

We present a comparative study of the heterologous expression strains *Bacillus megaterium*, *Escherichia coli* HMS174(DE3), *Shimwellia blattae* and a commercial strain of *Vibrio natrigenes*, for cobalamin class-III dependent enzymes expression. The *Nitratireductor pacificus pht-3B* reductive dehalogenase (NpRdhA) and the epoxyqueuosine reductase from *Streptococcus thermophilus* (StoQ) were used as model enzymes. We also analysed whether co-expression of the cobalamin transporter *BtuB*, supports increased cobalamin incorporation into these enzymes in *E. coli*. We conclude that while expression in *Bacillus megaterium* resulted in the highest levels of cofactor incorporation, co-expression of *BtuB* in *E. coli* presents an appropriate balance between cofactor incorporation and protein yield in both cases.

## Highlights

• Reductive dehalogenases and epoxyqueuosine reductases are cobalamin-dependent.
• Cofactor incorporation presents a challenge for heterologous expression of these enzymes.
• A range of bacterial hosts were assessed as expression host for these enzymes.
• An inverse correlation between protein yield and cofactor content was observed.
• Co-expression with the BtuB B12 transporter in *E. coli* yielded best results.

## 1 Introduction

The widespread use of anthropogenic organohalides as solvents, pharmaceuticals and feedstock chemicals for synthesis is well documented [1]. However, their large-scale synthesis, coupled with improper disposal, has resulted in contamination at a number of sites. Organohalides are often toxic and can be highly carcinogenic compounds that are recalcitrant to degradation due to the strength of the carbon halogen bond. As a consequence, clean up of organohalide-contaminated sites presents a serious challenge [2,3]. A diverse group of bacteria has been shown to selectively degrade a variety of organohalides. Under anaerobic conditions, certain bacteria use organohalides as terminal electron acceptors for an energy conserving process called organohalide respiration (OHR) and are therefore known as organohalide respiring bacteria (OHRB) [4]. The OHR process is reliant on reductive dehalogenases (RDases) that reductively cleave carbon halogen bonds and thus have obvious potential for bioremediation [5].

**Fig. 1:**
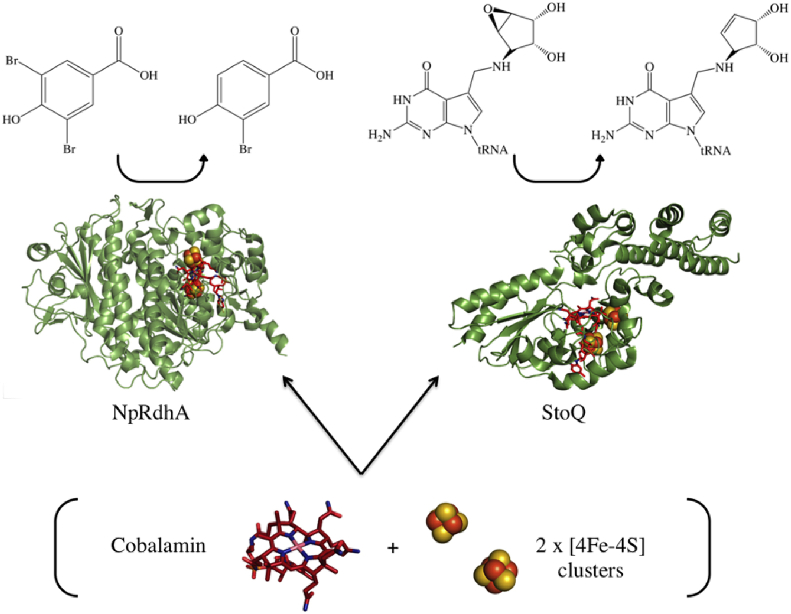
***S. thermophilus* Epoxyqueuosine reductase (StoQ) and *N. pacificus pht-3B* Reductive dehalogenase (NpRdhA*)*-** Both NpRdhA and StoQ are cobalamin class-III dependent enzymes, containing a central cobalamin and 2 [4Fe–4S] clusters. Both catalyse very different reactions with NpRdhA (PDB: 4RAS) reductively cleaving carbon halogen bonds (shown via dehalogenation of 3,5-dibromo-4-hydroxybenzoic acid to 3-bromo-4-hydroxybenzoic acid) and StoQ (PDB: 5D6S) involved with reduction of the epoxide moiety in the modified nucleotide epoxyqueuosine, to queuosine.

RDase enzymes form the class-III family of cobalamin containing enzymes, alongside the distantly related epoxyqueuosine reductase (QueG), that contain a central cobalamin cofactor bound by a nitroreductase-like fold and 2 [4Fe–4S] clusters [6,7]. Unlike the RDases, who harness their cobalamin cofactor for the cleavage of carbon halogen bonds, the QueG enzymes are involved in the biosynthesis of the modified nucleotide queuosine, found ubiquitously in eukaryotes and bacteria, in the wobble position of anticodon tRNA molecules containing the 5′-G_34_U_35_N_36_-3′ anticodon sequence (Fig. 1). The heterologous expression and purification of QueG from *Bacillus megaterium* and *Escherichia coli* has been reported previously, with the latter devoid of cofactors and requiring reconstitution [6,[8], [9], [10]].

Unlike its QueG counterparts, expression and purification of RDases has been a recurring struggle, with limited success in purification directly from the host organisms [[11], [12], [13]]. This is due to the often impractical requirement for poorly water-soluble organohalides for growth, resulting in slow growth and low cell biomass [14]. Further more, heterologous expression of RDases (often with solubility enhancing tags), in standard heterologous expression strains such as *E. coli*, resulted in soluble but catalytically inactive protein [15,16]. Recently successful cases of heterologous expression of catalytically active RDases have been reported, such as the *Nitratireductor pacificus pht-3B* reductive dehalogenase (NpRdhA) expressed in *B. megaterium* [17] and the *Sulfurospirillum multivorans* perchloroethene reductive dehalogenase expressed in *Shimwellia blattae* [18], both bacterial hosts being able to perform *de novo* biosynthesis of cobalamin [[19], [20], [21]].

The complete *de novo* biosynthesis of vitamin B12, or cobalamin in its biological form, requires over 30 enzyme-mediated reactions occurring by either an anaerobic or aerobic pathway [[21], [22], [23]]. However, due to the complexity of biosynthesis, the large energy requirement involved and the relatively small cellular requirement for cobalamin, a number of organisms have “opted out” with only prokaryotes and some archaea able to perform cobalamin biosynthesis. These organisms tightly regulate this process, both by translational and transcriptional control, presenting a challenge to cobalamin incorporation into heterologously expressed RDases [24,25].

Many organisms use a salvage system allowing the transport of extracellular cobalamin into the cytoplasm instead. This process is again under strict translational control, with *E. coli* utilizing a cobalamin riboswitch that prevents translation of *BtuB*, the initial cobalamin transporter across the periplasmic membrane, in cobalamin rich conditions [26].

We set out to compare a number of commonly used heterologous expression systems, *S. blattae, E. coli* HMS174(DE3)*, B. megaterium* and a commercial strain of *Vibrio natrigenes,* Vmax™, for the expression of class-III cobalamin-dependent enzymes, in order to determine their relative merits. We selected Terrific Broth (TB) as a nutritionally rich bacterial medium for high cell growth density and kept growth conditions as well as protein purification protocols constant for all strains. Expression of Vmax™ was also performed in an optimal, enhanced 2xYT medium. In addition, we study the effect of co-expressing the *E. coli* cobalamin transporter, BtuB on the levels of cobalamin incorporation [27,28]. The reductive dehalogenase from *N. pacificus pht-3B* (NpRdhA) [17] and the epoxyqueuosine reductase from *S. thermophilus* (StoQ) [8] are used as model enzymes. We identify trends in pure protein yields, cobalamin and [4Fe–4S] cluster incorporation levels, suggesting future strategies for expression and purification of class-III cobalamin-dependent enzymes.

## 2 Methods

### 2.1 Molecular biology

All proteins of interest were cloned allowing for the production of protein containing a C- or N-terminal hexahistidine tag for the *N. pacificus* pht-3B reductive dehalogenase (NpRdhA, **WP_008597722.1**) and *S. thermophilus* epoxyqueuosine reductase (StoQ, **WP_011227267.1**) respectively. The expression vectors used for Vmax™ and *E. coli* HMS174(DE3) cell lines were pET30 and pET28a for NpRdhA and StoQ respectively, each of which are under the control of a T7 promoter, allowing induction of expression by addition of isopropyl-β-d-1-thiogalactopyranoside (IPTG). Co-expression of *E. coli* BtuB (**WP_000591352.1**) was performed in pET3a using the same conditions described for HMS174(DE3).

The vectors pN-His-TEV1622 [29] and pPT7 [30] were used for expression of StoQ and NpRdhA respectively in *B. megaterium* allowing protein induction via a xylose promoter. The vector pASK-IBA63c-plus (IBA Lifesciences, Germany) was used for protein expression in *S. blattae,* which allowed induction by a tetracycline promoter. NpRdhA and StoQ inserts were PCR amplified using the appropriate primers (Fig. S1) using CloneAMP HiFi PCR Premix (Takara). Template StoQ and NpRdhA can be seen in Figs. S2 and S3 respectively. PCR products were cloned into the desired vector using Infusion HD enzyme mix (Clontech) and transformed into *E. coli* Stellar cells. Constructs were confirmed by DNA sequencing before the purified plasmid underwent transformation into the desired host strain.

### 2.2 Transformation protocols

Mineral media *B. megaterium* protoplast transformations were performed as described in Ref. [31]. Transformation of *S. blattae* cells was performed as described in Ref. [32]. Both the commercial strain of *V. natrigenes* (Vmax™ express cells), and HMS174(DE3) were transformed using the manufacturer's recommendations [33], and [34] respectively.

### 2.3 Heterologous expression

All cell lines were grown in a Type NLF 22, 30 L BioEngineering fermenter containing 22 L Terrific Broth (Formedium) at 37 °C. Once at an OD_600_ of 1 the temperature was reduced to 18 °C and the media supplemented with 1 μM vitamin B12 and 50 μM ammonium iron (II) sulphate for overnight protein induction. Expression was induced with 1 mM IPTG for Vmax™ and HMS174(DE3) cell lines. *B. megaterium* expression was induced with 0.1% xylose. *S. blattae* expression was induced with 20 ng/mL anhydrotetracycline (hydrochloride) anaerobically under a continuous nitrogen gas flow of 30 L/min^−1^.

In addition to growing Vmax™ cells in TB medium, cells were also cultured in enhanced 2xYT medium (20 g yeast extract, 32 g tryptone, 17 g NaCl, 0.2% glucose, 17.6 mM Na_2_PO_4_ pH 7.4 per litre). Heterologous expression was induced as in TB medium, however induction temperature remained at 30 °C.

Average wet weight cell mass recovery can be seen in Fig. S4 and were in the range of 5–24 g/L. Lower values resulted from *S. blattae* probably as a consequence of anaerobic induction.

### 2.4 Protein purification

StoQ and NpRdhA containing cell pellets (60 g from individual fermenter growths) were re-suspended in lysis buffer (100 mM NaPi pH 7.5, 400 mM NaCl and 50 mM Tris pH 7.5, 200 mM NaCl respectively) each with DNase (Sigma) and EDTA-free protease inhibitor tablets (Roche) and for NpRdhA pellets also with RNase (Sigma). The cells were lysed using a cell disruptor (Constant Cell Disruption Systems, Daventry, UK) at 20 Kpsi and then lysate cleared by centrifugation in a Beckman Coulter Optima L-100 XP ultracentrifuge at 185000 × *g* for 1 h at 4 °C. Cell lysate volume was measured for total protein quantity analysis before purification was performed. StoQ and NpRdhA purification methods differed from here onwards.

StoQ cleared lysate was applied to a 5 mL HiTrap HP column (GE Healthcare) using a P-1 peristaltic pump (GE Healthcare) at 4 °C after which the column was attached to an ÄKTA pure purification system. The column was washed with 5-column volumes (CV) lysis buffer after which a linear gradient of 0–500 mM imidazole in lysis buffer was performed over 35 CV. Fractions containing StoQ were collected, concentrated using a 30 kDa molecular weight cut-off Vivaspin (GE Healthcare) spin column centrifugal concentrator, in a Sigma 3-16 PK centrifuge fitted with an 11180 rotor at 3894 × *g* and 4 °C. Imidazole was removed using a 10 mL CentriPure P100 Zetadex gel filtration column (EMP Biotech). StoQ was loaded according to the manufacturer's instructions and eluted using 50 mM Tris pH 7.5, 200 mM NaCl after which, purified protein concentration was determined by UV–visible absorbance spectroscopy at 280 nm.

NpRdhA clarified lysate was applied to a 10 mL Ni-NTA agarose drip column (Qiagen) pre-equilibrated with lysis buffer at 4 °C. The column was then washed with lysis buffer containing 15 mM and 30 mM imidazole (4 CV of each) and protein was eluted with 20 mL of 250 mM imidazole. Similarly to StoQ, eluted protein was concentrated and imidazole removed by gel filtration using 50 mM Tris pH 7.5, 200 mM NaCl.

Protein purity was judged by applying samples to a BioRad, Mini-Protean TGX stain free precast SDS-gel with a 4–20% gradient and were visualized using a BioRad, Gel Doc™ EZ Gel Documentation System.

### 2.5 UV–visible spectroscopy/purified protein quantification

UV–visible absorbance spectra were recorded using a Cary 50 UV–Vis spectrophotometer. Purified protein concentration and total protein yield were estimated for StoQ and NpRdhA using the extinction coefficients *ε*_280_ = 48,360 M^−1^ cm^−1^ and *ε*_280_ = 77,810 M^−1^ cm^−1^ respectively (calculated from the respective primary amino acid sequence using the ProtParam program on the ExPASy proteomics server) and the final volume of purified protein.

### 2.6 Total soluble crude extract protein quantification

Total soluble protein content from the crude extract supernatant was determined using the Biuret assay. Biuret reagent was prepared by dissolving 0.75 g CuSO_4_·5H_2_0 and 3 g sodium potassium tartrate in a final volume of 500 mL 3 M sodium hydroxide. Briefly, 50–100 μL clarified crude extract was diluted up to a final volume of 1 mL in water. Samples were mixed with 4 mL Biuret reagent and left to stand for 20 min at room temperature after which time the sample was measured at 550 nm on a Cary 50 UV–Vis spectrophotometer. The absorbance of the buffer blank sample was subtracted from the protein test sample and multiplied by the dilution factor to give the protein concentration.

### 2.7 Folin Ciocalteu protein quantification

Purified protein concentrations were determined by a modification of the method described in Ref. [35]. A standard curve of 0–100 μg/mL BSA was prepared in 1 mL with distilled water. The protein of interest and the buffer the protein was resuspended in were diluted to 1 mL so that the estimated concentration of the protein was in the middle of the standard curve. A total of 5 mL Folin reagent (0.04 mM CuSO_4_, 0.02% (w/v) Na_2_CO_3_ in 0.1 M NaOH) was added to each sample and left to incubate at room temperature for 15 min. A volume of 0.5 mL of Folin and Ciocalteu's reagent (diluted 1:1 with distilled water) was added and mixed by vortexing. After 35–45 min the absorbance was recorded at 750 nm and the protein concentrations calculated from the standard curve after subtracting the buffer blank and adjusting for the dilution factor used.

### 2.8 Spinach ferredoxin and *E. coli* flavodoxin reductase driven NpRdhA activity analysis

Activity analysis was performed using either crude extract or 1 μM purified NpRdhA under anaerobic conditions at 30 °C. Reactions were set up in a glove box (Belle Technology, UK) under an N_2_ environment and contained 5 mM 3,5-dibromo-4-hydroxybenzoic acid, 10 mM NADPH, 100 μM spinach ferredoxin and 10 μM E*. coli* flavodoxin reductase. Reactions were sealed within 2 mL amber crimp-top HPLC vials and incubated for 30 min [2]. The reactions were stopped by the addition of 6% final concentration trichloroacetic acid, centrifuged at 14100 × *g* to remove precipitated protein and analysed using an HPLC.

### 2.9 HPLC analysis

HPLC analysis was performed on an Agilent 1260 Infinity HPLC with a UV diode array detector attached. The stationary phase used was a Kinetex® 5μ C18 100 Å column, 250 × 4.6 mm. The mobile phase was water/acetonitrile (50:50) containing 0.1% trifluoroacetic acid at flow rate of 1 mL min^−1^ for 10 min.

### 2.10 Electron paramagnetic resonance spectroscopy

Samples were prepared for EPR after concentration of protein to ~30 mg/mL in 50 mM Tris pH 7.5, 200 mM NaCl. Samples were transferred into 4 mm Suprasil quartz EPR tubes (Wilmad, USA) and directly frozen and stored in liquid nitrogen. EPR experiments were conducted using the parameters as follows: microwave power 0.5 mW, modulation frequency 100 kHz, modulation amplitude 5 G, temperature 30 K. These parameters are non-saturating for the EPR signals concerned. Spectra were obtained using a Bruker ELEXSYS E500 spectrometer, Super high Q resonator (ER4122SHQ), Oxford Instruments ESR900 cryostat and ITC503 temperature controller.

### 2.11 Iron and cobalamin quantification

In order to extract iron from the protein for analysis, the protein was mixed with an equal volume of 2 M HCl and heat denatured at 80 °C for 10 min, followed by removal of precipitate by centrifugation. A suitable quantity of the sample (10–200 μL) was taken for assay with bathophenanthroline using the method previously described in Refs. [2,8,17] before measurement of the absorbance at 535 nm. Iron concentrations were determined from an iron standard curve over the range 0–50 nmol.

Cobalamin concentration was estimated after extraction by dicyano-complex formation or via spin-quantification of the Co(II) signal using EPR. Given the ligand-dependent oxidation-reduction equilibria of cobalamin, all cobalamin present in the sample is cob (II)alamin under the conditions employed.

Cyanide extraction involved mixing of 0.4 mM protein and potassium cyanide (10 mM) followed by heating of the sample in a fume hood for 20 min at 80 °C. Measurement of the cyanocobalamin UV–visible spectrum and quantification of concentration using the 550 nm reading (*ε* = 8.7 mM^−1^ cm^−1^) allowed determination of the protein bound cobalamin concentration.

Cobalamin quantification via EPR was performed as described in Ref. [17] using a 1 mM Cu-EDTA standard run under exactly the same conditions as the sample of interest.

## 3 Results

### 3.1 Protein purification comparison

**Fig. 2:**
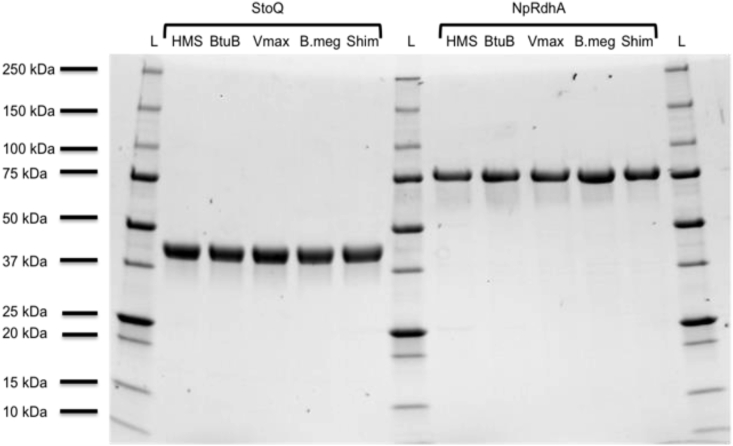
***S. thermophilus* Epoxyqueuosine reductase (StoQ) and *N. pacificus pht-3B* reductive dehalogenase (NpRdhA*)* purified from different expression strains-** Lanes annotated as follows: HMS= HMS174(DE3), BtuB = HMS174(DE3) co-transformed with BtuB, Vmax = Vmax™ express cells, Bmeg = *B. megaterium,* Shim = *S. blattae.* Theoretical StoQ size = ~45.1 kDa. Theoretical NpRdhA size = ~77.7 kDa. Protein content was standardized before loading.

Cloning for both NpRdhA and StoQ, into strain specific expression vectors, was performed to incorporate a C- or N-terminal hexahistidine tag respectively. An optimized one-step purification method was developed for each enzyme. Heterologous expression and one-step purification of StoQ (~45 kDa, Fig. S5) and NpRdhA (~78 kDa, Fig. S6) were performed in the various expression strains tested, in duplicate, resulting in >95% purity for all proteins as judged by SDS-PAGE (Fig. 2).

In an attempt to standardize expression conditions, TB was used as the expression medium for all strains given previous use for the expression of both NpRdhA and StoQ [2,8]. However, for protein expression in Vmax™, the recommended medium is an enhanced 2xYT media (2xYT), with induction at 30 °C [36], which was also tested alongside a TB based protocol. In the case of *S. blattae* previous studies report the use of minimal medium under anaerobic conditions [18]. However, under these conditions no protein expression was seen for StoQ or NpRdhA (data not shown). Furthermore protein yield was only measured in soluble fractions due to solubilised pellet fractions showing consistently less than 10% target protein.

Table 1 shows StoQ purified protein yields obtained from *S. blattae* (StoQ^Shim^, 180 ± 6 mg) were highest, and *B. megaterium* (StoQ^Bmeg^, 14 ± 1 mg) the lowest. Vmax™ cells grown in TB medium (StoQ^Vmax−TB^) and *E. coli* HMS174(DE3) (StoQ^HMS^) cells both produced similar quantities of StoQ (57 ± 3 mg and 63 ± 7 mg respectively). In comparison to HMS174(DE3), HMS174(DE3) cells co-expressing BtuB (StoQ^BtuB^) resulted in twice as much soluble StoQ (130 ± 15 mg). The quantity of StoQ from Vmax™ cells grown in 2xYT medium was increased when compared to TB conditions (86 ± 7 mg and 57 ± 3 mg, respectively).

**Table 1:** Protein content and cofactor analysis of StoQ- Total soluble protein was determined using the Biuret reagent. Final purified protein was determined by UV–visible spectroscopy. B12 values show cobalamin content determined via EPR with cyanide extraction based values shown in parenthesis. All purifications performed in duplicate using 60 g of wet weight cell mass from individual fermenter growths.

Expression Strain	Total Soluble Protein (mg)	Final Purified Protein (mg)	B12 Occupancy (%)	Fe:Protein content
Vmax™ express (TB)	3300 ± 340	57 ± 3	24 (18)	7.5 ± 0.3
*Bacillus megaterium*	1800 ± 14	14 ± 1	90 (83)	8.0 ± 0.1
HMS174(DE3)	2500 ± 64	63 ± 7	62 (46)	5.2 ± 0.1
HMS174(DE3) + BtuB	3700 ± 260	130 ± 15	70 (52)	5.3 ± 0.1
*Shimwellia blattae*	5000 ± 270	180 ± 6	12 (7)	7.2 ± 0.6
Vmax™ express (2xYT)	4500 ± 190	86 ± 7	51 (35)	5.7 ± 0.1

Similar trends are observed for NpRdhA in TB medium, Table 2. On average, the quantity of NpRdhA obtained from expression in *S. blattae* (NpRdhA^Shim^, 220 ± 52 mg) was highest and the least from *B. megaterium* (NpRdhA^Bmeg^, 11 ± 1 mg). Co-expression of BtuB again resulted in a two fold increase in soluble protein in comparison to standard HMS174(DE3) cells (NpRdhA^BtuB^, 160 ± 12 mg and NpRdhA^HMS^, 75 ± 10 mg, respectively). Contrasting with StoQ, the quantity of NpRdhA from Vmax™ in TB medium (NpRdhA^Vmax−TB^, 24 ± 4 mg) was two thirds lower than NpRdhA^HMS^. NpRdhA purified from Vmax™ grown in 2xYT medium conditions (NpRdhA^Vmax−YT^) produced a ~7.7 fold increase in NpRdhA purified, in comparison to NpRdhA^Vmax−TB^ (180 ± 50 mg and 24 ± 4 mg respectively). Excellent yields of NpRdhA^BtuB^, NpRdhA^Vmax−YT^ and NpRdhA^Shim^ were therefore obtained, demonstrating the importance of both expression strain as well as expression conditions.

**Table 2:** Protein content, cofactor and activity analysis of NpRdhA- Total soluble protein was determined using the Biuret reagent. B12 values show cobalamin content determined via EPR with cyanide extraction based values shown in parenthesis. Activity analysis was performed using both crude extract and purified protein using a non-cognate reductase system composed of Spinach ferredoxin (100 μM) and *E. coli* flavodoxin reductase (10 μM) under anaerobic conditions. Purified protein activity/B12 was determined using average B12 content to determine the active site concentration. Recovery was determined using total activity of crude extract and purified protein. All purifications were performed in duplicate using 60 g of wet weight cell mass from individual fermenter growths.

Expression Strain	Total Soluble Protein (mg)	Final Purified Protein (mg)	B12 Occupancy (%)	Fe:Protein content	Crude Extract Specific Activity	Purified Protein Specific Activity	Purified Protein Activity/B12	Recovery (%)
Vmax™ express	2500 ± 85	24 ± 4	25 (24)	7.7 ± 0.1	18 ± 1.1	180 ± 8	720 ± 33	10.0 ± 1.4
*Bacillus megaterium*	1600 ± 110	11 ± 1	38 (37)	6.5 ± 0.1	4.1 ± 0.1	290 ± 15	780 ± 15	48.0 ± 4.3
HMS174(DE3)	2300 ± 670	75 ± 10	21 (19)	3.6 ± 0.5	24 ± 0.8	170 ± 3	830 ± 53	25.0 ± 10.0
HMS174(DE3) + BtuB	2300 ± 300	160 ± 12	30 (26)	5.4 ± 0.2	35 ± 0.2	200 ± 8	720 ± 68	38.0 ± 7.6
*Shimwellia blattae*	5000 ± 740	220 ± 52	8 (3)	4.1 ± 0.1	13 ± 0.3	130 ± 7	ND*	42.0 ± 3.7
Vmax™ express (2xYT)	6700 ± 160	180 ± 50	38 (30)	6.3 ± 0.2	20 ± 0.3	260 ± 11	770 ± 120	35.0 ± 8.5

*ND-not-determined due to high degree of uncertainty in exact B12 content. Crude extract and purified protein specific activity = μM product min^−1^ mg^−1^; Purified protein activity/B12 = μM product min^−1^ mg ^−1^/B12 concentration.

### 3.2 StoQ and NpRdhA cobalamin content

**Fig. 3:**
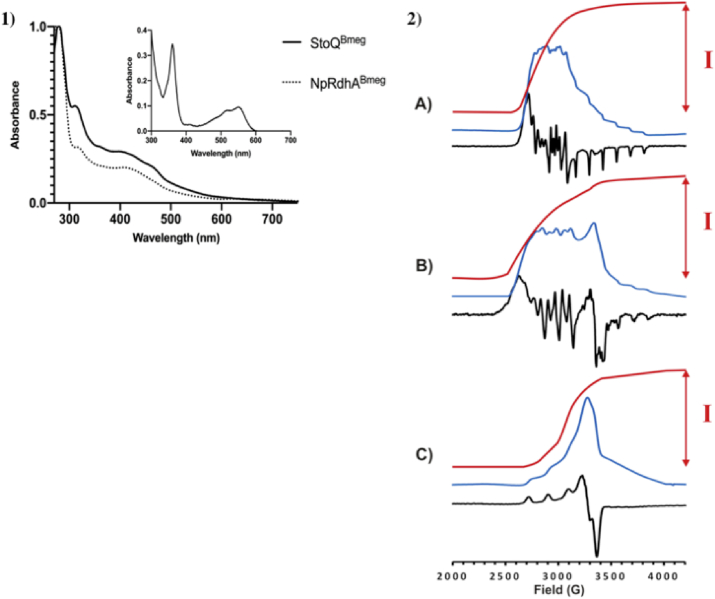
**Spectroscopy for cobalamin quantification-** 1) UV–visible spectra of StoQ and NpRdhA purified proteins. Spectra were recorded on a Cary 50 UV–Vis spectrophotometer from wavelengths of 200–800 nm. Inset shows a typical cyanocobalamin spectrum observed from cobalamin extraction using cyanide, from both StoQ and NpRdhA. 2) Estimation of cobalamin content using X-band EPR. Recorded spectra (black), first integral of that spectrum (the absorption spectrum, blue), and ‘double’ integrals (red) of A) 200 μM StoQ; B) 200 μM NpRdhA; C) 1 mM Tris-Cu^2+^. I indicates the value of the double integral that is proportional to the number of Cu^2+^ ions and Co^2+^-containing cobalamin molecules detected in the experiment. Given the known concentration of Cu^2+^ ions in that sample and the known protein concentrations of StoQ and NpRdhA in the samples, the number of cobalamin molecules per mole protein can be determined by ratio. Spectra are not shown on same vertical scales.

Levels of StoQ and NpRdhA cobalamin incorporation were determined by both EPR spin quantification of the Co(II) signal at 30 K and UV–visible spectroscopy quantification using cobalamin cyanide extraction (Fig. 3) [17]. Before quantification, StoQ and NpRdhA were concentrated to approximately 30 mg/mL to allow accurate determination of cobalamin incorporation (Table 1, Table 2). Exact protein concentration was determined using the Folin Ciocalteu reagent [35].

With respect to StoQ, cobalamin incorporation was highest for StoQ^Bmeg^ (90%), presumably due to the ability of *B. megaterium* to synthesize cobalamin *de novo*. StoQ^HMS^ with and without co-expression of BtuB contain the next highest cobalamin incorporation, with the co-expressing BtuB strain resulting in a slightly higher cobalamin content than HMS174(DE3) alone (70 and 62% respectively). The cobalamin incorporation in StoQ^HMS^ is higher than expected, showing the high efficiency of BtuB and its ability to transport cobalamin, even while under translational control [37,38]. In contrast, both the protein purified from Vmax™ and *S. blattae* contained substantially less cobalamin.

Similar trends are seen for NpRdhA (Table 2), with NpRdhA^Bmeg^ containing the most cobalamin (38%) and NpRdhA^Shim^ the least (non-detectable). NpRdhA^BtuB^ has higher cobalamin incorporation than NpRdhA^HMS^ (30 and 21% respectively). In contrast with StoQ cobalamin incorporation, NpRdhA^Vmax−TB^ has marginally increased cobalamin (25%) than NpRdhA^HMS^.

In the case of Vmax™ expression, the proteins obtained from 2xYT have increased cobalamin incorporation alongside increased purified protein yields in comparison to the TB based protocol. StoQ^Vmax−TB^ and StoQ^Vmax−YT^ had 24% and 51% cobalamin incorporation respectively, whilst NpRdhA^Vmax−TB^ and NpRdhA^Vmax−YT^ showed 25% and 38% respectively. Interestingly NpRdhA^Vmax−YT^ cobalamin incorporation level was comparable to that seen with NpRdhA^Bmeg^. In both cases, the HMS174(DE3)+BtuB strain presents a compromise between high cobalamin incorporation and sufficient protein yield.

### 3.3 Iron-sulphur cluster content

StoQ and NpRdhA both contain 2 [4Fe–4S] clusters [8,17]. Determination of iron:protein ratios reveals StoQ^Vmax−TB^, StoQ^Shim^ and StoQ^Bmeg^ all have very similar iron content (7.5 ± 0.3, 7.2 ± 0.6 and 8.0 ± 0.1 iron:protein respectively; Table 1). This equates to 1.9 ± 0.1, 1.8 ± 0.2 and 2.0 ± 0.1 [4Fe–4S] clusters per StoQ monomer, suggesting a full complement of [4Fe–4S] clusters. In contrast, StoQ^HMS^ (both with and without BtuB) has a much lower iron:protein ratio (iron:protein ratio of 5.3 ± 0.1 and 5.2 ± 0.1 respectively), indicating inefficient cluster incorporation in this case. StoQ^Vmax−YT^ also had a lower iron:protein ratio than StoQ^Vmax−TB^ (5.7 ± 0.1 and 7.5 ± 0.3 respectively).

The iron-content for purified NpRdhA appears generally lower compared to StoQ (Table 2). NpRdhA^Vmax−TB^, NpRdhA^Vmax−YT^ and NpRdhA^Bmeg^ have high iron:protein ratios of 7.7 ± 0.1, 6.3 ± 0.2 and 6.5 ± 0.1 respectively, indicating the presence of 1.9 ± 0.1, 1.6 ± 0.1 and 1.6 ± 0.1 [4Fe–4S] clusters per NpRdhA, respectively. In contrast, NpRdhA^Shim^ has a low iron:protein ratio of 4.1 ± 0.1, suggesting only 1 [4Fe–4S] cluster per NpRdhA. As observed with StoQ, NpRdhA^HMS^ (both with and without BtuB) has a low iron:protein ratio of 5.4 ± 0.2 and 3.6 ± 0.5 respectively. The [4Fe–4S] cluster content for NpRdhA is increased when co-expressed with BtuB (NpRdhA^BtuB^, 1.4 ± 0.1 versus 0.9 ± 0.2). The cobalamin incorporation has previously been implicated in NpRdhA iron-sulphur cluster maturation, due to the close contact between cobalamin and the proximal [4Fe–4S] [17].

### 3.4 NpRdhA reductase specific activity is strain dependent

We were unable to routinely determine StoQ activity due to difficulties in obtaining sufficient amounts of the tRNA substrate. However, the NpRdhA 3,5-dibromo-4-hydroxybenzoic acid (3,5-DB-4-OH) reductase activity can readily be determined, making use of a non-cognate redox system (consisting of *E. coli* flavodoxin reductase and spinach ferredoxin) to support the transfer of electrons from NADPH to the NpRdhA [2,17]. Activity measurements were performed on both crude extract and purified enzyme.

Crude extract activity from *B. megaterium* is by far the lowest of all the expression strains studied, with a specific activity of 4.1 ± 0.1 μM min^−1^mg^−1^, corresponding with the small quantity of NpRdhA purified from these cells. However, upon purification of NpRdhA^Bmeg^, the activity increased to 290 ± 15 μM min^−1^mg^−1^, the highest of any strain tested, showing the quality of NpRdhA^Bmeg^ protein.

Despite the undetectable levels of cobalamin, *S. blattae* has crude extract activity of 13 ± 0.3 μM min^−1^mg^−1^ and a specific activity of 130 ± 6.7 μM min^−1^mg^−1^. *S. blattae* therefore has the lowest specific activity out of all the strains studied, reflecting the cofactor incorporation observed with this strain under the growth conditions used.

As described previously, cobalamin incorporation into StoQ and NpRdhA derived from HMS174(DE3) is increased when co-expressed with the *E. coli* transporter BtuB. This increase in cobalamin incorporation is reflected in the NpRdhA 3,5-DB-4-OH reductase activity, which is higher in NpRdhA^BtuB^ than NpRdhA^HMS^, for both the crude extracts (35 ± 0.2 and 24 ± 0.8 μM min^−1^mg^−1^, respectively) and purified protein (200 ± 8 and 170 ± 3 μM min^−1^mg^−1^, respectively).

Surprisingly, the 3,5-DB-4-OH reductase activity in NpRdhA^Vmax−TB^ crude extract was much higher than expected (18 ± 1.1 μM min^−1^mg^−1^), considering the quantity of NpRdhA purified from these cells. However, NpRdhA^Vmax−TB^ crude extract activity was lower than NpRdhA^Vmax−YT^ (20 ± 0.3 μM min^−1^mg^−1^) due to the 7.7 fold increase in purified NpRdhA under the 2xYT conditions. The quality of both purified NpRdhA^Vmax−TB^ and NpRdhA^Vmax−YT^ was further revealed by 3,5-DB-4-OH reductase activity (180 ± 8 and 260 ± 11 μM min^−1^mg^−1^ respectively) reflecting the increase in cofactor incorporation under 2xYT media expression conditions.

When calculating specific 3,5-DB-4-OH reductase activity using the protein bound concentration of cobalamin (Table 2, Activity/B12 (μM product min^−1^mg^−1^/B12)) reveals a remarkably consistent specific activity for all samples, confirming cobalamin content as the key variable. Specific activity could not be obtained for NpRdhA^Shim^, due to a non-detectable cobalamin level.

## 4 Discussion

The heterologous expression and one-step purification of both model enzymes StoQ and NpRdhA yielded active protein, albeit to a varying degree of success. Indeed, using a TB medium and standardized protein purification method, yielded distinct quantities of each protein for the various expression strains tested. In both cases, *B. megaterium* gave the lowest yield while *S. blattae* the highest. However, an apparent trade off between the quantity of the protein purified and the quality of this protein, in terms of cofactor incorporation, is observed. In this case, *B. megaterium* supports the highest cobalamin incorporation, which is reflected in the 3,5-DB-4-OH reductase activity of NpRdhA purified from this strain. Cobalamin incorporation in StoQ^Bmeg^ was similar to that previously reported (i.e. 0.7 ± 0.1 per monomer [8]) however was much reduced in NpRdhA^Bmeg^ compared to previous levels reported (i.e. 0.82 per monomer [17]). This suggests that some NpRdhA bound cobalamin is lost during the aerobic one-step purification protocol used in this report.

Although *S. blattae* produces the highest quantity of StoQ and NpRdhA, cobalamin incorporation is low (12% and non-detectable respectively). The lack of *holo*-protein formation in *S. blattae* could be attributed to its ability to synthesize pseudo-cobalamin, rather than cobalamin, that both StoQ and NpRdhA require [18]. Another potential explanation could be due to the quantity of protein produced, causing problems with folding and cofactor incorporation into StoQ and NpRdhA from *S. blattae*. Attempts to optimise expression conditions in this strain may increase both [4Fe–4S] cluster and cobalamin incorporation.

Co-expression of class-III cobalamin-dependent enzymes with the cobalamin transporter BtuB in *E. coli* HMS174(DE3) may provide an acceptable compromise between protein yield and specific activity. Not only does this strain result in high protein yields, but also leads to relatively high cobalamin incorporation for both StoQ and NpRdhA (70 and 30% compared to 90 and 38% in *B. megaterium*, respectively). Unfortunately, the iron-sulphur cluster incorporation appears substantially lower when compared to other strains, which will require further study to improve incorporation further, potentially by co-expression of the iron-sulphur cluster pathway operon [39,40].

In an attempt to standardize expression conditions, Vmax™ cells were grown in suboptimal conditions. Upon expression in a recommended enhanced 2xYT medium, both quantities of purified StoQ and NpRdhA and cobalamin incorporation increased in comparison to when grown in TB, therefore showing the importance of not only expression strain utilized, but also conditions under which they are expressed. A potential explanation for the increased protein obtained and cobalamin incorporation could be due to the large quantity of NaCl present in the enhanced 2xYT medium. NaCl has proved essential for optimal growth and also protein synthesis in *V. natrigenes*, therefore potentially explaining both the increased purified StoQ and NpRdhA obtained under these conditions, as well as cobalamin incorporation due to increased expression of salvage systems [[41], [42], [43]]. The importance of expression conditions was further demonstrated by the lack of StoQ and NpRdhA expressed in *S. blattae* (data not shown), under conditions previously successful for expression of other RDases [18].

## 5 Conclusion

In this study we have used two model class-III cobalamin-dependent enzymes, StoQ and NpRdhA, to test a range of heterologous expression hosts for their ability to produce the corresponding holo-enzymes in high yield. Neither isolation directly from the OHRB host, or reconstitution of the apo-protein have been reported as efficient procedures that can yield the required protein amounts to support detailed biochemical and biophysical studies on the mechanism and structure of these enzymes. When keeping most variables, in terms of protein purification and cell growth conditions, as similar as possible, we find that the cobalamin-producing *B. megaterium* offers the highest specific activity/level of cobalamin incorporation.

However, protein yield is modest compared to other strains tested under these conditions, and when striking a balance between yield and levels of cofactor incorporation, the BtuB expressing *E. coli* strain is the best candidate heterologous host for the expression of class-III cobalamin-dependent enzymes, on the basis of the two model enzymes tested.

Future work will need to focus on the study of additional members of the class-III enzymes to verify our findings. The NpRdhA enzyme is arguably distinct from respiratory reductive dehalogenases and appears not associated with the membrane. Furthermore, there are the so-called “self-sufficient” reductive dehalogenases, that are fused to the redox module that provides reducing equivalents from NAD(P)H, that provide an attractive target for future bioremediation. To date there are very few examples of these proteins having been expressed successfully and purified to homogeneity [44,45].

## Conflicts of interest

The authors declare that they have no conflict of interest with the contents of this article.

## Author contributions

T.H. and K.F. grew cells, purified protein and performed experiments. K. F. and S. E. J. R. carried out EPR spectra and analysed the EPR spectroscopic data. K.A.P.P. helped with molecular biology. D.L. conceived and coordinated the study. All authors wrote the paper, reviewed the results and approved the final version of the manuscript.

## Declaration of competing interest

The authors declare that they have no known competing financial interests or personal relationships that could have appeared to influence the work reported in this paper.
