# Supplementary material for: Heterologous expression of cobalamin dependent class-III enzymes
Source: Protein Expr Purif. 2021 Jan;177:105743. doi: 10.1016/j.pep.2020.105743 (PMC7585037; doi:10.1016/j.pep.2020.105743)
Supplement: Multimedia component 1 [file mmc1.docx]

**Supplementary Information**

| **Primer Name** | **Sequence (5’-3’)** |
| --- | --- |
| PET30-NP-F | AAGGAGATATACATATGCGCCTTTATTCCAATCG |
| PET30-NP-R | GGTGGTGGTGCTCGAGGCCAGCGCTAGATTTAAGACG |
| PET28a-StoQ-F | CGCGCGGCAGCCATATGAATATCAAACTGGAAATCCAGAAAATG |
| PET28a-StoQ-R | GGTGGTGGTGCTCGAGTTAAATCTGCCATTTCGCACAAACG |
| pPT7-NP-F | GGAGGTGAAATGTACAATGCGCCTTTATTCCAATCG |
| pPT7-NP-R | GGCCGGTACCGGATCCTTAGTGATGGTGATGGTGATGGCCAGCGCTAGATTTAAGAC |
| pNHIS-1622-StoQ-F | CAAGGCGCCCAGATCTCAATGAATATCAAACTGGAAATCCAGAAAATG |
| pNHIS-1622-StoQ-R | GGCCGGTACCGGATCCTTAAATCTGCCATTTCGCACAAACG |
| PASK-63c-NP-F | AGGAGATATACCATGCGCCTTTATTCCAATCG |
| PASK-63c-NP-R | TCCAAGCGCTCTCGAGTTAATGGTGATGGTGATGGTGGCCAGCGCTAGATTTAAGACG |
| PASK-63c-StoQ-F | AGGAGATATACCATGGTCCAAACTAGTTTCGAACATCACCATCACCATCACTCAGCTGGTGAAAACTTATATTTCCAAGGCGCCCAGATCTCCATGAATATCAAACTGGAAATCCAGA |
| PASK-63c-StoQ-R | TCCAAGCGCTCTCGAGTTAAATCTGCCATTTCGCACAAACGA |

**Figure S1. Primers used in this study-** Primers were designed to allow the creation of constructs enabling heterologous expression of StoQ or NpRdhA, with an N- or C-terminal hexahistidine tag respectively, in the bacterial strains studied. PCR was performed using CloneAMP HiFi PCR Premix (Takara). PCR products were cloned into the desired vector using Infusion HD enzyme mix (Clontech) and transformed into *E. coli* Stellar cells. Constructs were confirmed by DNA sequencing before the purified plasmid underwent transformation into the desired host strain

ATGAATATCAAACTGGAAATCCAGAAAATGGCCAAAGAAATCGGGATTAGTAAAATTGGCTTTACAACGGCGGATGACTTCGACTACCTTGAGAAATCTCTGCGCCTTGGCGTCGAAGAAGGACGTACGACGGGTTTTGAGCACAAGAACATCGAGGAACGCATCTACCCGAAACTGTCATTGGAATCCGCGAAAACCATTATCAGCATTGCAGTAGCGTACCCGCATAAACTGCCTCAACAACCGCAGAAAACCGAGTTTAAACGCGGGAAAATTACCCCGAATAGCTGGGGCCTGGACTACCACTATGTGTTACAGGATAAACTGAAGCGCTTAGCAAAAGGCATCGAGAAATTAACCGAAAACTTCGAGTACAAAGGCATGGTAGATACTGGCGCGCTGGTGGACACTGCCGTTGCCAAACGTGCCGGAATTGGTTTCATTGGCAAAAATGGCTTGGTTATCTCGAAAGAATATGGATCCTATATGTATCTGGGTGAACTTATTACCAACCTGGAAATTGAGCCAGATCAGGAAGTCGATTATGGTTGCGGTGATTGTCGTCGCTGCTTGGATGCGTGTCCGACAAGCTGCCTGATTGGGGATGGGACAATGAATGCTCGTCGCTGCCTGTCGTTTCAGACCCAAGACAAGGGCATGATGGACATGGAATTTCGCAAGAAGATTAAAACTGTCATCTATGGCTGCGACATTTGCCAGATTAGCTGTCCGTATAATCGGGGTATTGACAATCCATTGGCATCAGATATCGATCCTGATCTTGCGATGCCAGAACTCCTGCCGTTTCTGGAACTGACGAACAAATCGTTCAAAGAAACCTTCGGTATGATCGCCGGTTCTTGGCGTGGTAAGAACATTCTGCAGCGTAACGCTATCATTGCTCTGGCCAATCTGCATGATCGGAACGCGATCGTGAAACTCATGGAAATTATCGACAAGAACAATAACCCCATTCATACCGCTACGGCGATTTGGGCACTGGGTGAAATCGTGAAGAAACCCGATGAAGGCATGCTCGATTACATGCGCGGCTTAAGTCCGAAAGATGAGCACAGCCAAGCAGAATGGGAGCTCGTTTGTGCGAAATGGCAGATTTAA

**Figure S2. StoQ template DNA**

ATGCGCCTTTATTCCAATCGTGATCGCCCTAACCACCTGGGTCCACTTGCCCTTGAACGCCTTGCCCGTGTTGATGATGTTGTAGCGCAACCAGCACGTCAGCCGGAAGATGGCTTTGCAGCTTCCGAAGATTCTCTGTTAGGAGATGTAGAAGAATATGCTCGTTTATTTACGCGCTTTCTTGATGGTCCAGTCGCGCCGTTAGGCGATGCCATTCCTGATGATCCAGCACGTCGCGCTGAAAATCTTAAAGCGAGCGCCTACTTTCTGGATGCATCAATGGTTGGTATTTGCCGTTTAGATCCGGATGATCGCGCTGGCGATTGTGATCCGAGTCATACTCACGCATTAGTATTTGCTGTCCAATTTGGCCGCGAACCGGAAGCTGGTGAAGCAGGCGCTGAATGGATTCGTGGAACAAACGCAGCACGTACCGATATGCGTTGCGCAGAAATTGCAGCTATCCTGTCTGGATATGTTCGCTGGATGGGTTTTCCTGCACGTGGTCATTTTAGCGGCGATGCTCAGGTTGATCTGGCACGTTTAGCAGTGCGTGCAGGACTGGCTCGCGTTGTGGATGGAGTTCTGGTGGCACCATTTTTACGTCGCGGATTTCGCTTAGGTGTAGTCACAACCGGATATGCACTTGCAGCAGATCGTCCACTGGCACCTGAAGGAGATTTAGGTGAAACGGCACCGGAAGTGATGCTGGGCATTGATGGAACTCGTCCTGGATGGGAAGATGCTGAAGAAGAAAAACGTCCTTTACACATGGGTCGCTATCCAATGGAAACCATTCGTCGCGTGGATGAACCGACGACTCTGGTTGTGCGCCAAGAAATCCAGCGTGTAGCGAAACGCGGCGATTTCTTTAAACGTGCGGAAGCCGGTGATTTAGGCGAAAAAGCGAAACAAGAAAAGAAACGTTTTCCTATGAAACACCCATTAGCCCTTGGAATGCAACCGTTAATTCAGAATATGGTACCTCTTCAGGGAACACGCGAAAAACTGGCTCCTACCGGTAAAGGTGGCGATCTGAGCGATCCAGGCCGTAACGCAGAAGCTATTAAAGCGTTAGGCTATTACCTTGGAGCCGATTTTGTTGGTATCTGCCGTGCAGAACCTTGGATGTATTACGCTAGCGATGAAGTGGAAGGCAAACCAATTGAAGCATATCATGATTACGCTGTAGTCATGTTAATCGATCAAGGATATGAAACGATGGAAGGAGCTTCAGGTGATGATTGGATTTCAGCAAGTCAGTCCATGCGTGCTTATATGCGCGGCGCGGAAATCGCCGGAGTTATGGCAGCTCATTGTCGTCGCATGGGTTACAGTGCGCGTTCTCATAGCAATGCCCACTCCGAAGTTATTCACAACCCTGCGATCCTGATGGCCGGCTTAGGAGAAGTGTCCCGCATTGGAGATACACTTCTGAATCCGTTTATTGGTCCTCGTTCAAAAAGTATCGTTTTTACAACCGATCTTCCAATGTCTGTGGATCGTCCGATTGATTTTGGCCTGCAAGATTTTTGTAACCAGTGCCGTAAATGTGCGCGCGAATGTCCGTGCAATGCCATCTCATTTGGTGATAAAGTGATGTTTAACGGCTATGAAATCTGGAAAGCGGATGTAGAAAAATGCACGAAATACCGCGTCACTCAAATGAAAGGTTCAGCATGCGGCCGCTGTATGAAAATGTGTCCATGGAATCGTGAAGATACGGTAGAAGGTCGTCGCCTTGCAGAACTGAGTATTAAAGTCCCGGAAGCTCGTGCGGCCATTATCGCAATGGATGATGCTCTTCAGAACGGAAAACGTAACCTTATCAAACGTTGGTGGTTTGATTTAGAAGTAATCGATGGAGTCGCAGGAGCACCACGTATGGGTACAAATGAACGCGATCTGAGTCCGGATCGTGGAGATAAAATTGGTGCGAACCAAAAACTGGCCATGTATCCGCCTCGCTTACAGCCACCGCCTGGTACGACTCTTGATGCGGTTCTGCCGGTGGATCGTTCTGGTGGTCTGGCCGAATACGCAGCTGCAGAAACCCCTGCAGCAGCTCGTGCACGTCTTAAATCTAGCGCTGGCTAA

**Figure S3. NpRdhA template DNA**

| **Expression Strain** | **StoQ average cell mass (g)** | **StoQ cell mass (g/L)** | **NpRdhA average cell mass (g)** | **NpRdhA cell mass (g/L)** |
| --- | --- | --- | --- | --- |
| Vmax^TM^ express (TB) | 410 | 19 | 520 | 24 |
| *Bacillus megaterium* | 420 | 19 | 300 | 14 |
| HMS174(DE3) | 220 | 10 | 180 | 8 |
| HMS174(DE3) + BtuB | 180 | 8 | 220 | 10 |
| *Shimwellia blattae* | 160 | 7 | 100 | 5 |
| Vmax^TM^ express (2xYT) | 390 | 18 | 530 | 24 |

**Figure S4. Wet weight cell mass recovery-** Heterologous expression of both StoQ and NpRdhA was performed in a Type NLF 22, 30 L BioEngineering fermenter containing 22 L Terrific Broth or an enhanced 2 YT medium (2xYT). Expression culture was harvested by centrifugation at 6000rpm for 10 minutes at 4°C in a Beckman Coulter, Avanti J26-XP centrifuge fitted with a JLA 8.1000 rotor and cell mass weighed.

**Figure S5. Representative purification of StoQ using the developed method-** A) SDS-PAGE gel of a typical StoQ purification using a 5ml HisTrap HP column. L=load, FT=flow through, W=wash with lysis buffer after application of the column to the ÄKTA, 1-10= elution fractions used to asses fraction contents produced via a gradient from 0-500 mM Imidazole in lysis buffer over 35 column volumes. B) Vmax^TM^ StoQ purification elution profile from ÄKTA pure. Green line represents concentration of B equating to 0-500 mM imidazole.


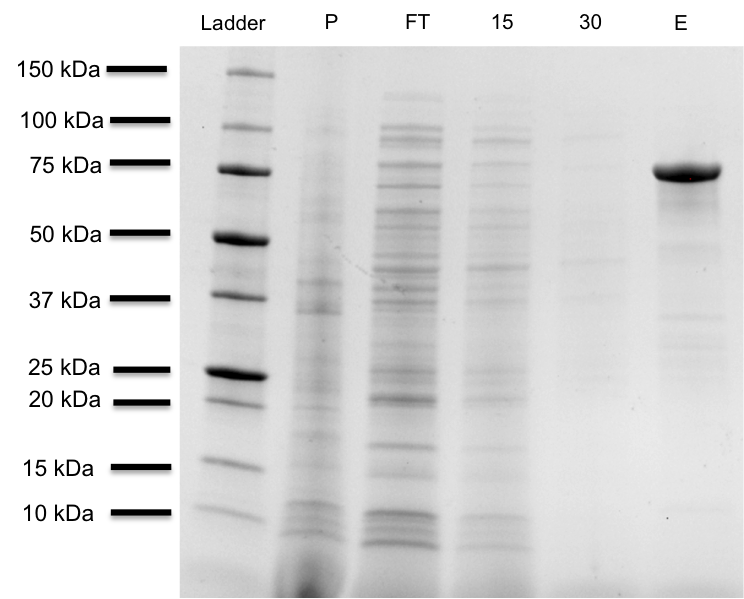


**Figure S6. Typical gravity flow Ni-NTA purification of NpRdhA-** Cells resuspended in lysis buffer (50 mM Tris (pH 7.5) 200 mM NaCl) and lysed via cell disruption. Protein purified at 4°C. P-pellet, FT = flow through of cleared lysate, 15 mM= wash with lysis buffer+15 mM imidazole, 30mM= wash with lysis buffer+30 mM imidazole, E= elution fraction using lysis buffer+250 mM imidazole
